# Evaluation of an implementation strategy for Individual Placement and Support in the Netherlands: a 30-month observational study

**DOI:** 10.1186/s12888-022-04121-9

**Published:** 2022-07-15

**Authors:** Miljana Vukadin, Frederieke G. Schaafsma, Harry W. C. Michon, Bart Cillekens, Peter M. van de Ven, Trees Juurlink, Johannes R. Anema

**Affiliations:** 1 Department of Public and Occupational Health, Amsterdam UMC, Vrije Universiteit Amsterdam, Amsterdam Public Health Research Institute, Van der Boechorststraat 7, NL-1081 BT Amsterdam, The Netherlands; 2 Research Centre for Insurance Medicine, collaboration between AMC, UMCG, UWV, VUmc, Amsterdam, The Netherlands; 3 Trimbos Institute, The Netherlands Institute of Mental Health and Addiction, Da Costakade 45, 3521 VS Utrecht, The Netherlands; 4 Movisie, Churchilllaan 11, 3527 BG Utrecht, The Netherlands; 5 Department of Epidemiology and Data Science, Amsterdam UMC, Vrije Universiteit Amsterdam, Amsterdam Public Health Research Institute, Van der Boechorststraat 7, NL-1081 BT Amsterdam, The Netherlands

**Keywords:** Severe mental illness, Supported employment, Implementation, Employment outcomes

## Abstract

**Background:**

Individual Placement and Support (IPS) is an evidence-based, effective approach to help people with severe mental illness (SMI) obtain and maintain competitive employment. The aim of the present study was to examine employment outcomes and associations with an organizational and a financial factor in people with SMI who participated in Individual Placement and Support using a multifaceted implementation strategy (IPS + MIS). The goal of this strategy was to improve IPS implementation by enhancing collaboration among mental health care and vocational rehabilitation stakeholders, and realizing secured IPS funding.

**Methods:**

An observational cohort study including 103 participants was conducted, with a 30-month follow-up. Descriptive analyses were used to examine employment outcomes. Multivariable logistic and linear regression analyses were performed to study associations with an organizational and a financial factor: the level of experience of mental health agencies with providing IPS + MIS and the type of IPS funding (i.e. municipality funding (reference group) and the Dutch Social Security Institute: the Institute for Employee Benefits Schemes (UWV) funding).

**Results:**

Forty-six percent of the participants were competitively employed at any time during the 30-month follow-up; the median number of days until competitive job obtainment and in competitive jobs was 201 and 265, respectively. The majority of all jobs obtained (81%) were categorized as ‘elementary occupations’, ‘clerical support workers’, and ‘service and sales workers’. A higher level of experience of the mental health agencies with providing IPS + MIS was found to be positively associated with job obtainment (OR = 3.83, 95% CI 1.42–10.30, *p* = 0.01) and the number of days worked in competitive jobs (B = 1.21, 95% CI 0.36–2.07, *p* = 0.01). UWV funding was found to be negatively associated with job obtainment (OR = 0.30, 95% CI 0.11–0.77, *p* = 0.01). No association was found for the type of IPS funding and the number of days worked in competitive jobs (B = -0.73, 95% CI -1.48–0.02, *p* = 0.06).

**Conclusions:**

This study shows that almost half of the people who participate in IPS + MIS obtain a competitive job within 30 months. The results further suggest that both the level of experience of mental health agencies with providing IPS + MIS, and funding may play a role in employment outcomes.

## Background

Obtaining and maintaining competitive employment is challenging for people with severe mental illness (SMI). Although most of these people have a wish to work [1–3], their employment rates are low [4, 5] and they often receive social assistance or disability benefits [6].

Individual Placement and Support (IPS) is a clearly described model of vocational rehabilitation and includes the following eight principles: eligibility based on client choice, focus on competitive employment, integration of mental health and employment services, attention to consumer choice, personalized benefits counselling, rapid job search, systematic job development, and time-unlimited and individualized follow-along support [7]. Research shows that IPS yields better employment outcomes than other vocational rehabilitation programs for people with SMI [8–10].

Although IPS is more effective than other programs, it is complicated to implement this model in both the Netherlands and other countries [11–18]. Inadequate funding and cooperation between mental health care and vocational rehabilitation services appear significant barriers to enhance further implementation in practice [4, 11–15, 17, 19–22]. To overcome these barriers, stakeholders from two mental health agencies, the Dutch Social Security Institute: the Institute for Employee Benefits Schemes (UWV), the municipality of Amsterdam, and a health insurance company in the Netherlands, initiated a collaboration. This collaboration consisted of a multifaceted implementation strategy (MIS), including an organizational and a financial component. The organizational component consisted of regular meetings among the different stakeholders involved, and the financial component consisted of secured IPS funding with a ‘pay for performance’ element. A detailed description of IPS using a multifaceted implementation strategy (IPS + MIS) within the context of this collaboration is provided in the methods section.

While the effectiveness of IPS has been documented in numerous studies [8–10], much less is known about the impact of contextual factors, including organizational and financial factors, on the implementation of IPS and its outcomes. Gaining more knowledge on these factors is considered important, as the outcomes of IPS are influenced by the quality of its implementation [23, 24].

Existing research mainly reports on the positive associations between IPS fidelity, reflecting the quality of IPS implementation, and employment outcomes [25–27]. A recent study, for example, has found that improved IPS fidelity is associated with improved employment outcomes over time [25], suggesting that the experience of mental health agencies with providing IPS could impact employment outcomes. However, it is possible that not only IPS fidelity, but also other factors, directly or indirectly influence the implementation of IPS and its outcomes [27, 28]. Funding, for example, is likely to indirectly influence the proportion of IPS participants commencing employment, mediated through potential impacts on fidelity [28].

Strategies aiming to improve IPS implementation in practice, such as the MIS, thus seem relevant, and need to be further explored. Since the goal of the MIS was to remove organizational and financial barriers [13, 14], it is likely that this strategy may help improve IPS implementation and employment outcomes of people with SMI.

Up to now, research on IPS + MIS has focused on stakeholders’ experiences with IPS + MIS [13, 14]. However, no studies have examined the employment outcomes of people with SMI who participated in IPS + MIS, and associations with an organizational and a financial factor related to this MIS. These two factors are 1) the level of experience of mental health agencies with providing IPS + MIS and 2) the type of IPS funding. Therefore, the objectives of this study were:

1. To examine employment outcomes of people with SMI who participated in IPS + MIS during the 30-month follow-up period.
2. To study associations between employment outcomes in people with SMI who participated in IPS + MIS and 1) the level of experience of mental health agencies with providing IPS + MIS and 2) the type of IPS funding.

This study contributes to the literature by providing a better understanding of what impact contextual factors may have on employment outcomes of people with SMI.

## Methods

### Study design

This was an observational cohort study conducted between 2014 and 2020 in people with SMI who participated in IPS + MIS.

### Context information

#### IPS + MIS within the context of the collaboration among stakeholders

Clients with SMI who received treatment at one of the two participating mental health agencies and expressed a wish to work to their mental health clinician, were referred to an IPS employment specialist of the same specialized mental health treatment team for an intake. These clients also received disability benefits from UWV or social assistance benefits from the municipality, or were not entitled to benefits.

During the intake phase, the IPS employment specialist and the client decided within eight consultations whether IPS was the right intervention for the client. Then the IPS employment specialist discussed in a multidisciplinary meeting with vocational rehabilitation practitioners of UWV and the municipality whether the IPS applicant qualified for funding, and if so, for which type of funding. This meeting and the funding were part of the multifaceted implementation strategy (MIS), which consisted of an organizational and a financial component [13, 14].

The organizational component comprises regular meetings at two levels among the professionals of the different organizations involved: 1) at the management level, there were regular meetings among decision makers who had a managing or advising role within their organization. Their main goal was to facilitate practitioners and ensure IPS sustainment; 2) at the practitioner level, there were regular meetings among IPS employment specialists and several vocational rehabilitation professionals. Their main goal was to organize the IPS funding for new clients, and to provide improved benefits counselling as compared to usual IPS practice [13, 14].

The financial component was composed of secured IPS funding with a pay for performance element, rewarding the participating mental health agencies with an additional fee for placing an IPS client in a competitive job. A fair or good IPS fidelity score was a condition for this funding [13, 14].

In the Dutch context, municipalities provide social assistance benefits and are responsible for vocational rehabilitation of clients who either receive these benefits or who are not entitled to any type of benefits; UWV is a national agency which provides disability benefits to its clients and is responsible for their vocational rehabilitation; UWV clients all paid in the past premiums as employees for this social security insurance. There were thus two types of IPS funding, that were based on the same principles, but the duration and amount of the funding depended on the type of benefits clients received [13, 14]:

1. IPS funding from the municipality: for clients who received social assistance benefits from the municipality or who were not entitled to any type of benefits, the maximum duration of the funding was 18 months and the maximum amount was 2700 euro (excluding job coaching) in the first year of the collaboration (i.e. 2014). To stimulate a successful IPS trajectory, the mental health agencies received an extra 1800 euro when placing a client in a competitive job (i.e. pay for performance element) [13, 14]. After the first year, the maximum duration was extended to 24 months, and the maximum amount was 7500 euro (including job coaching) without a pay for performance element.
2. IPS funding from UWV: for clients who received disability benefits from UWV, the maximum duration of the funding was 36 months and the maximum amount was 4840 euro (excluding job coaching). To stimulate a successful IPS trajectory, the mental health agencies received an extra 1210 euro when placing a client in a competitive job (i.e. pay for performance element) [13, 14].

#### IPS fidelity

The 25-item IPS fidelity scale [29] was used to assess the quality of implementation; it evaluates the quality of staffing, organization and services for IPS programs. The total score ranges from 25 to 125, and the critical cut-off point for being recognized as IPS program is > 74. Fidelity reviews were conducted by two trained, external assessors according to protocol during a full-day visit at the two participating mental health agencies. The ratings were based on interviews, team meeting observations, and document reviews; the two assessors discussed any rating discrepancies until consensus ratings were reached [25]. The IPS model fidelity was evaluated at five time points during the study period (2014–2020). The two participating mental health agencies scored ‘fair’ (74–99 points) or ‘good’ (100–114 points) at the first measurement in 2014, and both improved their IPS fidelity steadily, scoring ‘exemplary’ (115–125 points) at the last measurement in 2019.

#### Participants

Recruitment took place between March 2014 and July 2017 at the community mental health care divisions targeted at adults with SMI of the two participating mental health agencies. Inclusion criteria were: having a SMI (i.e. a psychiatric disorder that requires care or treatment, for which coordinated care from professional care providers in care networks is indicated to realize the treatment plan; the disorder is accompanied with serious impairments in social and/ or societal functioning and is persistent over time; the impairment is the cause and result of the psychiatric disorder [30]), age between 18 and 65 years, participating in IPS + MIS, and willingness to give informed consent. Exclusion criteria were: competitive employment at study entrance and full-time hospitalization. IPS employment specialists informed their clients about the study and asked the clients who were eligible to participate. Written informed consent was obtained from all participants included in the study.

### Procedures

#### Data collection

Data were prospectively collected during a 30-month follow-up period through interviews with participants, consisting of several self-report questionnaires regarding demographic characteristics, mental health (Mental Health Inventory-5 [31]: consisting of five items, scored on a 6-point Likert scale), self-esteem (Rosenberg Self Esteem scale [32]: consisting of ten items, scored on a 4-point Likert scale) and employment. Interviews were face to face and took place at the participants’ mental health agency; the questionnaires were administered and filled out by M.V. or a trained research assistant, working in the field of public and occupational health. In addition, IPS specialists provided information about the participants’ diagnoses and employment status.

#### Ethical considerations

The Medical Ethics Committee of the Amsterdam University Medical Centre gave approval for the study. All procedures performed in this study were in accordance with the ethical standards of this institutional research committee and with the 1964 Helsinki declaration and its later amendments or comparable ethical standards.

### Measures

#### Level of experience of the mental health agencies with providing IPS + MIS

The moment of the participant’s inclusion in the study (i.e. the day baseline measures were collected) was considered as a proxy for the level of experience of the two mental health agencies with providing IPS + MIS. At the start of this study, these two agencies were relatively unexperienced with providing IPS + MIS. It is probable that the level of experience of these two mental health agencies with providing IPS + MIS increased over time, as reflected by their increasing IPS fidelity scores. It is therefore plausible that clients who started their IPS trajectory later in time, participated in a high-fidelity IPS + MIS program, provided by the more experienced mental health agencies. Accordingly, to study the association between the level of experience of the mental health agencies with providing IPS + MIS and employment outcomes, the total inclusion period (i.e. 10 March 2014–27 July 2017) was dichotomized in the first half of the total inclusion period, 10 March 2014–18 November 2015, and the second half of the inclusion period, 19 November 2015–27 July 2017 (i.e. the group of clients who were included in the second half of the total inclusion period, participated in IPS + MIS when the mental health agencies were considered to be more experienced with providing IPS + MIS).

#### Type of IPS funding

The duration and amount of the IPS funding depended on the type of benefits the participant received: clients who received social assistance benefits from the municipality or who were not entitled to benefits, received funding from the municipality; clients who received disability benefits from UWV, received funding from UWV. The type of benefits the participant was receiving at baseline was therefore considered as a proxy for the type of IPS funding. Accordingly, to study the association between the type of IPS funding and employment outcomes, the type of benefits at baseline were dichotomized in social assistance benefits from the municipality or no benefits, and disability benefits from UWV.

#### Competitive employment and employment outcomes

Competitive employment was defined as having a paid job against prevailing wages, in a company or organization in the regular labour market, not set aside for persons with a disability, that is, in an integrated work setting. Information was derived from interviews with participants at baseline and after 6, 18 and 30 months, and from employment records filled out every two months by their employment specialists. All collected data on competitive employment were used to categorize the jobs obtained during the study period, and to calculate employment outcomes.

For objective 1. the employment outcomes were: 1) the proportion of participants who obtained competitive employment, 2) the median number of days until first competitive job obtainment, 3) the median number of days worked in competitive jobs, and 4) the characteristics of the jobs participants obtained during the 30-month follow-up, including number of jobs, median hours worked per week and median number of days worked in a job per job category (International Standard Classification of Occupations 2008 [33]). For objective 2 the employment outcomes were: 1) job obtainment, defined as having worked in a competitive job yes or no for one day or more, and 2) the number of days worked in competitive jobs at any time during the 30-month follow-up.

#### Confounders

The covariates gender, age, educational level, work history (worked in past 5 years yes/ no) and mental health (Mental Health Inventory-5 [31]) at baseline were considered as confounders for the associations between employment outcomes and 1) the level of experience of the mental health agencies with providing IPS + MIS and 2) the type of IPS funding. These confounders were chosen based on literature on predictors of employment outcomes in people with a mental illness [34–36].

### Statistical analyses

Before analyses were conducted, data cleaning was performed. Employment outcomes during the 30-month follow-up period were summarized using descriptive statistics. To evaluate whether the level of experience of the two mental health agencies with providing IPS + MIS was associated with job obtainment, logistic regression analysis was used with job obtainment as the dependent variable. The association between the level of experience of the two mental health agencies with providing IPS + MIS and the number of days worked in competitive jobs was assessed in the subgroup of participants who were employed at any time during the 30-month follow-up; participants who were not competitively employed were not included in these analyses. Because the number of days worked in competitive jobs was skewed to the right, a log transformation was used before performing this analysis. To assess this association, linear regression analysis was used with the number of days worked in competitive jobs as the dependent variable. In both the logistic and linear regression analysis, the moment of the participant’s inclusion in the study was used as the independent variable; clients who were included in the study during the first half of the inclusion period were the reference group. Associations between the type of IPS funding and job obtainment, and the number of days worked in competitive jobs, were also assessed using logistic and linear regression analysis, respectively. In these analyses, clients who were receiving social assistance benefits from the municipality or no benefits at baseline were the reference group. Participants were not included in analyses if specific data on confounders or employment were missing. For all analyses, both a crude and an adjusted analysis (adjusted for all predefined confounders) were performed. For all analyses, a two-sided significance level of 5% was used and 95%-confidence intervals (CIs) for odds ratios (ORs) and regression coefficients (Bs) were calculated. All statistical analyses were performed using SPSS 26.0 (SPSS, Chicago, IL, USA).

## Results

### Baseline characteristics

A total of 103 participants were included in this study. The baseline characteristics are shown in Table 1; the number of participants per characteristic varies between 98 and 103 due to missing data. A total of 80 participants (78%) had a low or medium level of education and less than half of the participants (45%) worked competitively in the past five years. The majority of the participants received disability benefits from UWV (59%) and had a psychotic disorder (72%).

**Table 1:** Baseline characteristics of the participants^a^

Characteristics	All participants (*N* = 103)
Sex male, N (%)	67 (65)
Mean age in years (SD)	38.7 (9.7)
Married, N (%)	5 (5)
Living independently, N (%)	90 (87)
Born in the Netherlands, N (%)	71 (69)
Low and medium level of education, N (%)	80 (78)
Worked competitively in past 5 years, N (%)	44 (45) (*N* = 98)
Disability benefits, N (%)	60 (59) (*N* = 102)
Psychotic disorder, N (%)	74 (72)
Mean score MHI-5 (mental health) (SD), range 0–100	45.1 (9.4) (*N* = 100)
Mean score RSE (self-esteem) (SD), range 0–30	18.4 (4.8) (*N* = 101)

^a^N varies between 98 and 103 due to missing data. *N* = 103 when the N is not indicated in the table

### Employment outcomes

The employment outcomes during the 30-month follow-up are shown in Table 2. A total of 47 participants (46%) were competitively employed at any time during the 30-month follow-up, and 29 participants (28%) worked 6 months (183 days) or longer. In the subsample of competitively employed participants (*N* = 47), the median number of days until competitive job obtainment was 201 (6.6 months), and the median number of days worked in competitive jobs was 265 (8.7 months).

**Table 2:** Employment outcomes during the 30-month follow-up

Employment outcomes	All participants (*N* = 103)
Obtained competitive employment (%)	47 (46)
Median number of days until competitive job obtainment [IQR]^a^	200.5 [58.8–537.3]
Median number of days worked in competitive jobs [IQR]^b^	265.0 [93.0–512.0]

^a^Subsample of participants competitively employed, *N* = 46

^b^Subsample of participants competitively employed, *N* = 47

**Fig. 1:**
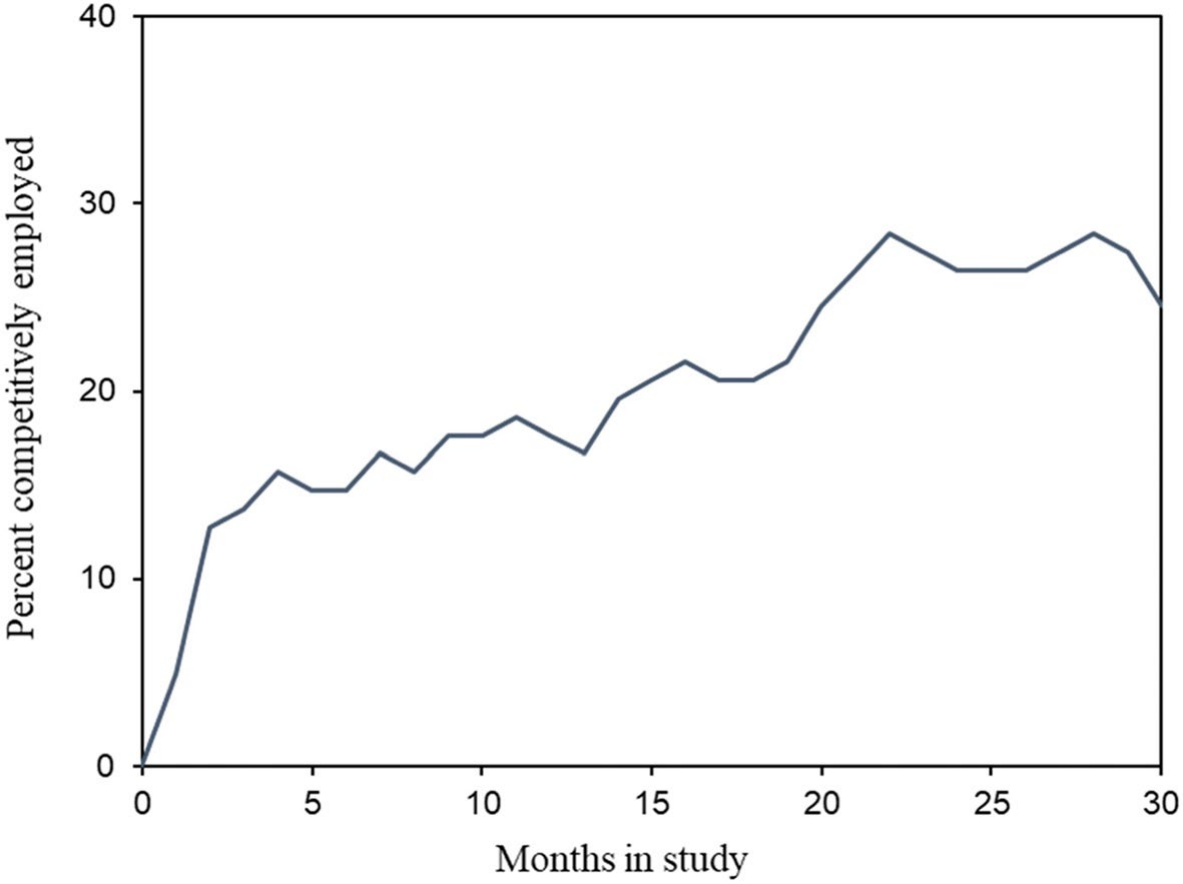
Monthly employment rates of the participants (*N* = 102) throughout the 30-month follow-up

Figure 1 presents the monthly employment rates of the participants (*N* = 102) throughout the 30-month follow-up. Participants rapidly obtained a rate of competitive employment of 13% by the second month of the study. Thereafter, their rate of competitive employment increased gradually to 28% in the 22th month and stabilized at around that level.

Table 3 presents the characteristics of the competitive jobs participants obtained during the 30-month follow-up period. All jobs (*N* = 73) were divided in seven categories; the categories ‘elementary occupations’ (*N* = 33, e.g. cleaner and shelf filler), ‘clerical support workers’ (*N* = 14, e.g. post carrier and customer contact centre information clerk) and ‘service and sales workers’ (*N* = 12, e.g. waiter and concierge) included the majority of all jobs obtained (81%). The overall median number of hours/week worked per job was 21, and the overall median number of days worked per job was 179 (5.9 months).

**Table 3:** Characteristics of the competitive jobs participants obtained during the 30-month follow-up period

Job category	All jobs (N)	Median hours/week worked per job^a^ [IQR]	Median number of days worked per job^b^ [IQR]
Professionals	2	12.5 [-]	194.0 [-]
Technicians and associate professionals	5	24.0 [14.0–24.5]	93.0 [77.5–496.0]
Clerical support workers	14	20.0 [15.5–28.0]	179.0 [45.0–360.0]
Service and sales workers	12	23.0 [17.0–30.0]	85.5 [14.3–229.5]
Plant and machine operators, and assemblers	4	13.0 [10.0–28.0]	420.0 [-]
Elementary occupations	33	20.0 [14.0–30.0]	210.0 [47.5–537.0]
Unknown job(s)	3	24.0 [-]	150.0 [-]
All job categories	73	21.0 [15.8–25.8]	179.0 [60.0–420.0]

^a^Based on 70 jobs due to missing data on total hours/week worked

^b^Based on 67 jobs due to missing data on total number of days worked

### Association between the level of experience of mental health agencies with providing IPS + MIS and employment outcomes

All results presented were adjusted for all the predefined confounders. The logistic regression analysis on job obtainment showed a statistically significant association between the moment of participant’s inclusion (proxy for the level of experience of the mental health agencies with providing IPS + MIS) and job obtainment (OR = 3.83, 95% CI 1.42–10.30, *p* = 0.01) (*N* = 95). Clients who were included in the study during the second half of the inclusion period were more likely to obtain employment compared to clients who were included in the study during the first half of the inclusion period. The linear regression analysis on the number of days worked in competitive jobs also showed a statistically significant association between the moment of participant’s inclusion and the number of days worked in competitive jobs (B = 1.21, 95% CI 0.36–2.07, *p* = 0.01, *R*-squared = 0.23) (*N* = 44). Clients who were included in the study during the second half of the inclusion period and obtained competitive employment, worked more days on average than the clients who were included in the study during the first half of the inclusion period and obtained employment.

### Association between the type of IPS funding and employment outcomes

All results presented were adjusted for all the predefined confounders. The logistic regression analysis on job obtainment showed a statistically significant association between receiving disability benefits at baseline (proxy for IPS funding from UWV) and job obtainment (OR = 0.30, 95% CI 0.11–0.77, *p* = 0.01) (*N* = 94). Clients who were receiving disability benefits from UWV at baseline were less likely to obtain employment compared to clients who were receiving social assistance benefits from the municipality or no benefits at baseline. The linear regression analysis on the number of days worked in competitive jobs did not show a statistically significant association between receiving disability benefits at baseline and the number of days worked in competitive jobs (B = -0.73, 95% CI -1.48–0.02, *p* = 0.06, *R*-squared = 0.15) (*N* = 44).

## Discussion

### Main findings

This study had two objectives: 1) examining the employment outcomes of people with SMI who participated in IPS + MIS, and 2) studying the associations between employment outcomes in these people and the level of experience of mental health agencies with providing IPS + MIS, and the type of IPS funding. Forty-six percent of the participants were competitively employed at any time during the 30-month follow-up. The competitively employed clients obtained their job within about 7 months, and worked for about 9 months in total. They obtained a variety of mostly entry-level jobs, such as elementary, clerical support, and service and sales positions. Furthermore, a higher level of experience of the mental health agencies with providing IPS + MIS was found to be positively associated with employment outcomes. In addition, IPS funding from UWV was found to be negatively associated with job obtainment, but not associated with the number of days worked in competitive jobs.

### Comparison with literature

In the present study, 46% of the IPS clients obtained a competitive job at any time during the 30-month follow-up. This percentage is in line with the percentages found in several previous European studies on IPS [37–40], reporting between 22% [40] and 46% [39] of the IPS clients being competitively employed during the follow-up. These studies [37–40], however, were randomized controlled trials, examining IPS implemented as usual, whereas the present study was an observational study focusing on IPS + MIS. The follow-up period was also longer in the present study than in some of the previous studies [38–40]. Although the labour market characteristics, policies and welfare systems differ between European countries [41], a recent meta-analysis suggests that these differences do not seem to impact employment outcomes [10].

Consistent with their educational level at baseline, participants in this study obtained mostly jobs that were categorized as ‘elementary occupations’, ‘clerical support workers’ and ‘service and sales workers’. This finding is in line with previous research [42, 43], which reports similar categories [43] and jobs [42], the majority of these jobs requiring a low or medium level of education.

Although no previous research studied the association between the type of IPS funding and employment outcomes in people who participated in IPS + MIS, several studies also suggest that funding is relevant for the implementation of IPS [4, 13, 14, 17, 20, 28], and therefore might influence employment outcomes [4, 17, 20, 25, 27, 28, 44].

### Strengths and limitations

This is the first European study examining the employment outcomes and associations with an organizational and a financial factor related to the MIS in people with SMI who participated in IPS + MIS. One of its strengths is the long follow-up period. Another strength is that the majority of data was collected through face to face interviews with IPS clients and that the employment outcomes were based on information provided by employment specialists, improving the quality of the results.

An important limitation is the assumption that the moment of participant’s inclusion is a proxy for the level of experience of mental health agencies with providing IPS + MIS. Although it is plausible that the differences in employment outcomes between the two groups of clients is due to the increased experience of the mental health agencies with providing IPS + MIS, it is also possible these differences are influenced by other factors, such as changes in economic conditions in the community, greater acceptance by community employers and legislation stimulating work participation [14]. Another limitation is the assumption that the type of benefits the participant was receiving at baseline was considered as a proxy for the type of IPS funding. Although this is a plausible assumption, the significant association between receiving disability benefits and job obtainment may have also been influenced by other factors, such as the difference in severity of the mental health problems and the distance from the labour market between the two groups of clients. Adjustment for work history and mental health in this analysis may have compensated this limitation somewhat. The sample size is also a limitation, since it was not possible to adjust for all potential confounders, such as diagnosis [36] and self-esteem [34]. To minimize selection bias, the employment specialists were asked to recruit all clients who met the inclusion criteria. However, it is possible that they recruited mostly motivated clients, causing selection bias. The employment specialists could also not provide more detailed information about all clients they had approached for IPS + MIS and/ or this study.

### Implications for practice and research

The low percentage of clients who worked 6 months or longer during the 30-month study period, deserves attention (only 28%). This percentage can be improved by helping more clients to obtain employment, and once employed, by helping them to stay longer employed. Since the IPS employment specialist’s role already seems prominent in helping clients to obtain employment [13], this low percentage suggests that employment specialists should focus more on helping clients maintaining their job. Based on literature, one way to accomplish this, could be improving the match between client and employer [45], for example, by improving employment specialists’ skills in job development (i.e. building an employer network by developing and maintaining relationships with various employers [46]) through training, repeated role-plays, field mentoring and group supervision [47]. Moreover, improved employment specialists’ skills in job development could also increase the probability of obtaining competitive employment [47–49]. Another way for employment specialists to help clients maintaining employment, could be with a more pro-active role when the client is employed, including more frequent contacts with the client, for instance at the job site [13, 50–52].

The finding that participants who were included later in time had better employment outcomes than participants who were included earlier in time, suggests that the experience of mental health agencies with providing IPS + MIS may indeed play a role in better employment outcomes. Stakeholders may gain more experience over time on how to collaborate successfully with each other (i.e. the organizational component of the MIS) and on how to organize more adequate funding (i.e. the financial component of the MIS), resulting in improved IPS services for clients. Stakeholders from mental health agencies who are planning to implement IPS + MIS in the near future, may benefit from collaborating with stakeholders from agencies who are experienced with providing IPS + MIS, as they can learn from their experience with organizing a collaboration among different organizations involved in IPS and funding [14, 17]. Since the IPS fidelity increased over time in both participating mental health agencies, this finding also suggests that the MIS may influence fidelity and thereby indirectly the proportion of IPS clients obtaining employment [25, 27, 28]. For mental health agencies that achieve high IPS fidelity, such as the mental health agencies in this study, focusing on improving other factors that can impact the implementation, may further improve employment outcomes [28]. Examples of organizational factors that deserve attention are lack of continuity due to a high staff turnover and lack of knowledge in newly hired staff [13, 14].

The finding that participants who received IPS funding from UWV were less likely to obtain employment than participants who received funding from the municipality, suggests that the duration and amount of IPS funding may play a role in employment outcomes of people who participated in IPS + MIS. One of the differences between the UWV and the municipality funding was the pay for performance element: the UWV funding included the pay for performance element during the first three years of the collaboration, while the municipality funding included it only during the first year of the collaboration. This suggests that the pay for performance element might not stimulate successful trajectories, as it probably does not influence IPS employment specialists in daily practice [13, 14]. Financing organizations could consider other types of financial incentives to improve outcomes, for example rewarding IPS employment specialists with monetary bonuses for placing clients in competitive jobs instead of the mental health agencies. However, future research is recommended to provide more insight into what impact funding has on employment outcomes. Furthermore, future research on the (cost-) effectiveness of IPS + MIS seems warranted, and should focus on employment outcomes related to maintaining competitive employment.

## Conclusions

This study shows that almost half of the people who participate in IPS + MIS obtain a competitive job within 30 months. The results further suggest that both the level of experience of mental health agencies with providing IPS + MIS, and funding may play a role in employment outcomes.

## Data Availability

The datasets generated and/or analysed during the current study are not publicly available due to privacy/ ethical restrictions but are available from the corresponding author on reasonable request.

## Abbreviations

IPS: Individual Placement and Support
MIS: Multifaceted implementation strategy
IPS + MIS: Individual Placement and Support using a multifaceted implementation strategy
SMI: Severe mental illness
UWV: The Dutch Social Security Institute: the Institute for Employee Benefits Schemes
